# High‐Efficiency Direct Ammonia Fuel Cells Based on BaZr_0.1_Ce_0.7_Y_0.2_O_3−_ *_δ_*/Pd Oxide‐Metal Junctions

**DOI:** 10.1002/gch2.201700088

**Published:** 2017-12-14

**Authors:** Yoshitaka Aoki, Tomoyuki Yamaguchi, Shohei Kobayashi, Damian Kowalski, Chunyu Zhu, Hiroki Habazaki

**Affiliations:** ^1^ Faculty of Engineering Hokkaido University N13W8 Kita‐ku Sapporo 060‐8628 Japan; ^2^ JST‐PRESTO 4‐1‐8 Honcho Kawaguchi 332‐0012 Japan; ^3^ Graduate School of Chemical Sciences and Engineering Hokkaido University N13W8 Kita‐ku Sapporo 060‐8628 Japan

**Keywords:** direct ammonia fuel cells, hydrogen membrane fuel cells, PCFC, RF sputtering deposition

## Abstract

A direct ammonia‐type intermediate temperature fuel cell is examined by means of a hydrogen membrane fuel cell (HMFC) comprising 1‐µm‐thick BaZr_0.1_Ce_0.7_Y_0.2_O_3−_ *_δ_* (BZCY) thin‐film electrolyte and Pd solid anode. It generates the maximum power density of 0.58 W cm^−2^ at 600 °C with ammonia fuels, and this value is found to be three times larger than the champion data of the recently reported direct ammonia‐type proton‐conducting ceramic fuel cells (PCFCs). AC impedance spectroscopy is performed to determine the interfacial polarization resistances, disclosing that the anodic overpotentials of HMFCs are at least one order of magnitude smaller than those of anode‐supported PCFC under relatively high DC outputs. The anode reactions are driven by the oxidation of monoatomic hydrogen dissolving at the BZCY/Pd solid–solid interface, mediated via proton transfer from Pd to BZCY. The electrochemical analysis reveals that the BZCY/Pd junction forms Ohmic contact without growth of wide depletion layer and thus facilitates the proton transfer reactions because the interfacial region beneath Pd electrode can accommodate amounts of protonic defects as well as the bulk of BZCY due to the small depletion of holes under hole–proton thermodynamic equilibrium.

## 1 Introduction

There is widespread consensus that the fossil fuel reserves will be exhausted to a large extent within next few decades due to accelerated growth of worldwide energy consumption. In the face of the energy crisis, hydrogen is attractive alternative fuel since it can be renewable by the water splitting driven with the natural power electricity without relying on fossil fuels, and thus a fierce development race is going on to make “clean hydrogen” economically and technologically viable. Advent of hydrogen economy, however, cannot be realized until technical issues related to hydrogen production, transportation, and a storage infrastructure are clear. Ammonia is a highly efficient hydrogen carrier since it can be easily liquefied to store and transport at room temperature, is widely available, is carbon‐free, and has hydrogen capacity larger than the liquid hydrogen.^1^, ^2^, ^3^, ^4^ Hence, it is strongly motivated to utilize ammonia in fuel cells for the direct conversion of ammonia chemical energy to electricity in a high efficiency. The proton conducting ceramic fuel cells (PCFCs) using proton‐conducting oxide electrolytes, such as BaZr*_x_*Ce_0.8−_ *_x_*Y_0.2_O_3−_ *_δ_*_,_ is promising as a direct ammonia type fuel cell operating at the intermediate temperature (IT) range (400–600 °C) for stationary applications.^4^, ^5^, ^6^, ^7^, ^8^, ^9^ Because water formation in PCFCs is mainly progressive at the cathode side, unlike solid oxide fuel cells (SOFCs), and therefore, mixing of the ammonia fuels into the exhaust water is precluded, thereby eliminating repurification of water exhaust and simplifying total systems. Moreover, formation of environmentally hazardous substances NO*_x_* at the anode is discouraged if the proton conductivity is much higher than the minor oxide ion conductivity.

**Figure 1:**
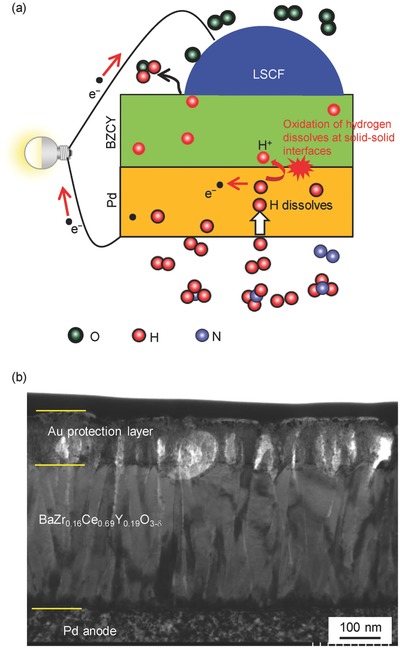
a) Scheme of a hydrogen membrane fuel cell (HMFC). b) Cross‐section TEM image of BaZr_0.1_Ce_0.7_Y_0.2_O_3–_ *_δ_* thin films (500 nm) sputter‐deposited on a Pd anode substrate.

Although the resistivity and related activation energy of proton conductors are smaller than oxide ion conductors used in SOFCs, the performances of the current PCFCs, however, are far behind the SOFCs even with pure H_2_ fuels because of large interfacial polarization due to a lack of suitable cathode^10^, ^11^, ^12^, ^13^, ^14^ and the deteriorated microstructural electrolyte.^15^, ^16^, ^17^, ^18^ Meanwhile, exceptionally high power output has been reported in the IT range for hydrogen membrane fuel cell (HMFC),^19^, ^20^, ^21^, ^22^, ^23^ which comprises a thin proton conducting ceramic electrolyte supported on a dense hydrogen permeable metal anode with the anode reactions driven by separation of the monoatomic hydrogen dissolves into protons and electrons at electrolyte/nonporous‐anode solid–solid interfaces (**Figure** **1**a). Regardless of its simple cell structure, the HMFC based on BaCe_0.8_Y_0.2_O_3_ (BCY) thin film and Pd foil has achieved the maximum power density of 1.4 W cm^−2^ at 600 °C,^19^, ^20^ which is still higher than the average performances of recently reported highly efficiency PCFCs at the temperatures.^24^, ^25^, ^26^, ^27^ Here, we report on the highly efficiency power generation of direct ammonia type HMFC with BaZr_0.1_Ce_0.7_Y_0.2_O_3−_ *_δ_* (BZCY) electrolyte due to enhanced proton incorporation into BZCY at the BZCY/Pd oxide‐metal junctions. The cell gained an open‐circuit voltage of about 1.0 V and maximum power density of 0.58 W cm^−2^ at 600 °C, which were much higher than the champion data of the recently reported direct ammonia type PCFCs.

## 2 Results

### 2.1 Fuel Cell Performances

BaZr_0.1_Ce_0.7_Y_0.2_O_3−_ *_δ_* films were successfully fabricated by RF cosputtering with BaCe_0.8_Y_0.2_O_3−_ *_δ_* and ZrO_2_ double targets in the deposition conditions listed in **Table** **1**. X‐ray diffraction patterns of deposited films were identical to BaCeO_3−_ *_δ_* phase (JCPDS 22‐0074) (Figure S1, Supporting Information), and the chemical composition determined by the wavelength dispersive X‐ray (WDX) analysis equaled Ba/Zr/Ce/Y = 1.0/0.16/0.69/0.19, which was in agreement with the objective one. The densely packed BZCY electrolytes thin films were uniformly formed over a wide area of the Pd foil anode without any microclacks and pinholes. The films have “bamboo” like microstructures, in which pillar grains of 10’s nm width are grown up perpendicular to the substrate and they are tightly adhered to each other. Such a columnar microstructure is frequently developed in the sputter‐deposited oxide films (Figure 1b).^28^, ^29^

**Table 1:** Optimal conditions for RF co‐sputtering deposition of BaZr_0.1_Ce_0.7_Y_0.2_O_3‐δ_ thin films

Conditions	
Sputtering atmosphere	Ar [50 cm^3^ min^−1^]
Substrate temperature	500 °C
Target‐substrate distance	70 mm
RF power	BaCe_0.8_Y_0.2_O_3_:70W, ZrO_2_:30W
Chamber pressure	2.0 Pa
Postannealing temperature	700 °C
Postannealing atmosphere	O_2_ [*p*_O2_ = 0.7 Pa]
Postannealing time	1 h

**Figure 2:**
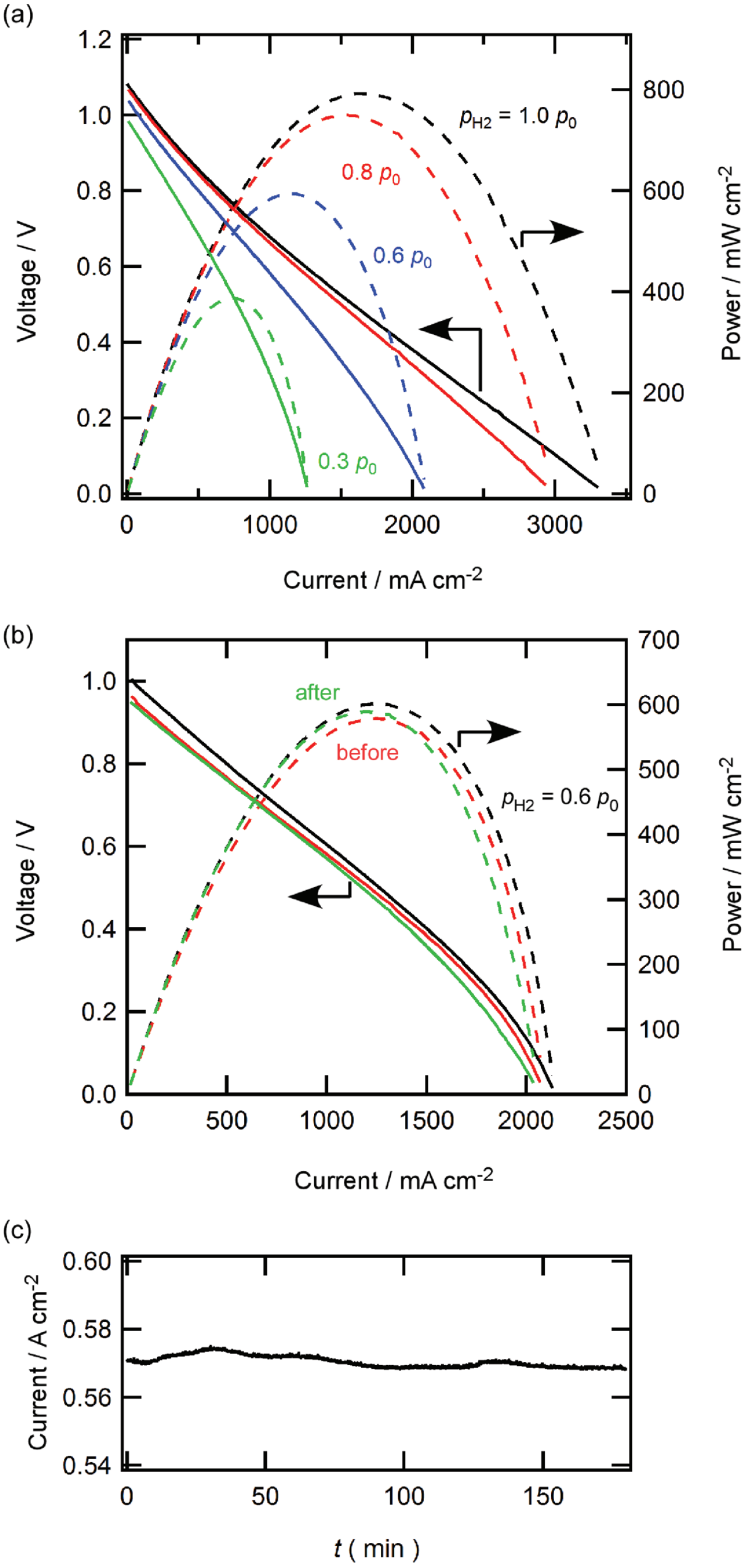
The current–voltage (*I*–*V*) and current–power (*I*–*P*) relationships of HMFC with a) H_2_ and b) NH_3_ fuels at 600 °C. a) *I*–*V* (——) and *I*–*P* (‐ ‐ ‐ ‐) characteristics of H_2_‐type HMFC operated at various hydrogen partial pressure (*p*_H2_) in the anode: H_2_/Ar (*p*_H2_ = 1.0–0.3 *p*_0_), Pd | BCY | LSCF, wet air. b) *I*–*V* (——) and *I*–*P* (‐ ‐ ‐ ‐) characteristics of direct ammonia‐type HMFC: NH_3_, Pd | BCY | LSCF, wet air. Red and green lines show performances before and after power generation at 0.75 V for 3 h, respectively. Performances of HMFC with a diluted H_2_ fuel at *p*_H2_ = 0.6 *p*_0_ are also shown as a reference (black). c) Current transients of direct ammonia type HMFC operating at a constant 0.75 V cell voltage for 3 h.

**Figure 3:**
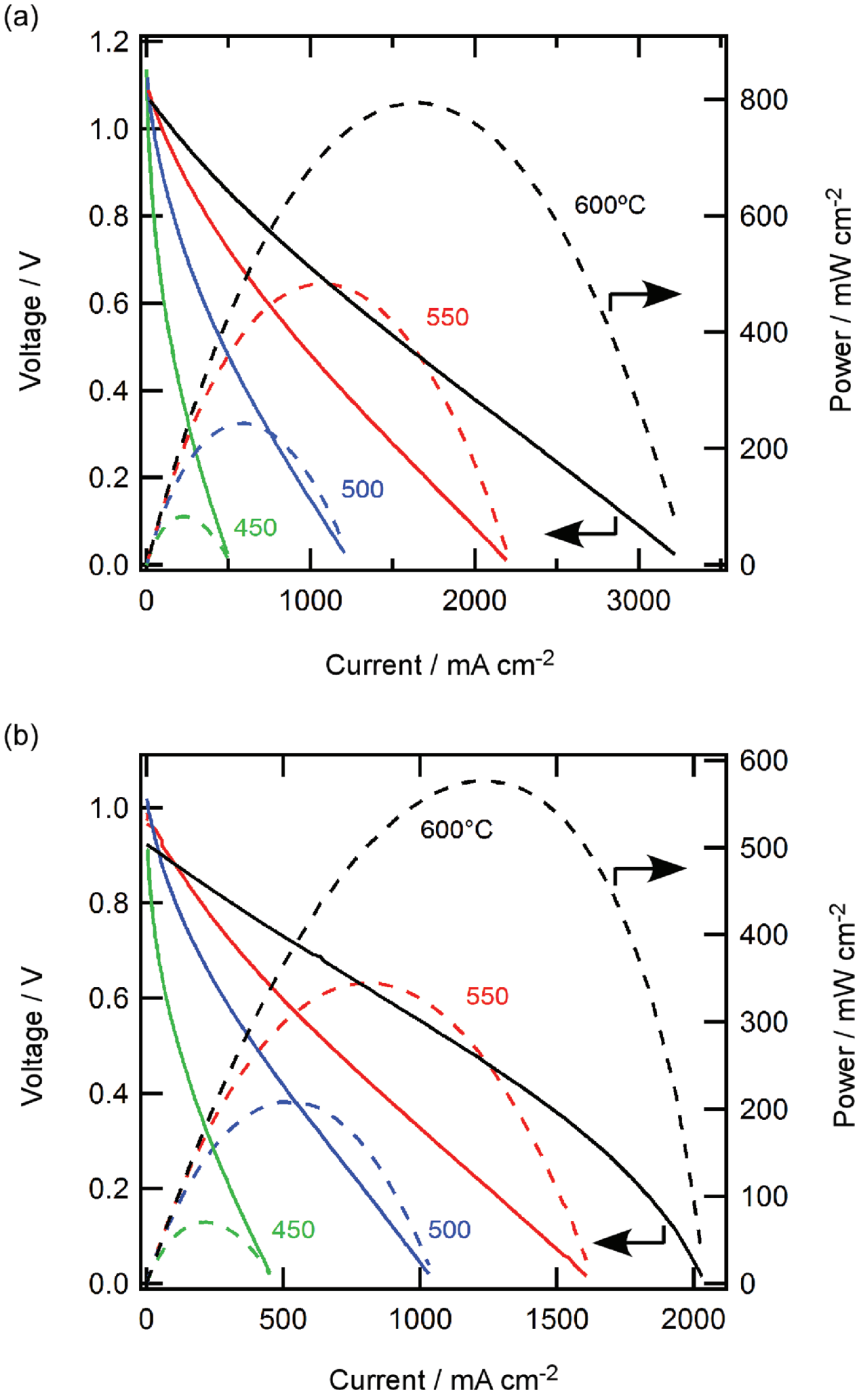
The current–voltage (*I*–*V*) and current–power (*I–P*) relationships of HMFC operating by feeds of a) H_2_ (*p*_H2_ = 1.0) and b) NH_3_ at various temperatures.

HMFC comprising a 1 µm thick BZCY thin film offers excellent cell performances at 600 °C, generating the peak power density (*P*_max_) of 0.81 W cm^−2^ and open‐circuit voltage (OCV) of 1.1 V with a pure H_2_ fuel (**Figure** **2**a). This value is similar to the values^24^, ^26^, ^27^ of highly efficiency PCFCs comprising from 10’s µm thick electrolytes and porous cermet anode supports. The HMFC retains OCV over 0.9 V if the hydrogen partial pressure (*p*_H2_) in the anode is decreased to 0.3 *p*_0_ (*p*_0_ = 101 kPa; Figure 2a). Moreover, the HMFC also enables highly efficient power generation by a direct use of NH_3_ fuels, providing OCV of 0.95 V and *P*_max_ of 0.58 W cm^−2^ at 600 °C (Figure 2b). The power output is very stable and the current remains at about 0.6 A cm^−2^ at 0.75 V for a few hours (**Figure** **3**c) without deterioration of the current–voltage (*I–V*) and current–power (*I–P*) characteristics (Figure 2b). It is warrant to note that the power output of NH_3_ type HMFC is identical to that of H_2_ type HMFC using H_2_ gases at *p*_H2_ = 0.6 *p*_0_ in the anode.

Figure 3 shows the *I–V* and *I–P* relationships of BZCY‐HMFC operating with H_2_ and NH_3_ fuels at various temperatures. The power outputs abruptly drop by decreasing the operation temperatures, with *P*_max_ gaining 0.81, 0.49, 0.24, and 0.085 W cm^−2^ at 600, 550, 500, and 450 °C, respectively for hydrogen fuel cells and *P*_max_ equaling 0.58, 0.34, 0.21, and 0.071 W cm^−2^ at 600, 550, 500, and 450 °C, respectively for ammonia fuel cells. The OCV of ammonia fuel cells would be increased with elevating temperatures if the following oxidation reaction of NH_3_ electrochemically takes place^30^, ^31^

(1) $${\mathrm{4NH}}_{3}\left(g\right)+{\mathrm{3O}}_{2}\left(g\right)\to {\mathrm{6H}}_{2}O\left(g\right)+{\mathrm{2N}}_{2}\left(g\right)$$

However, the actual OCV is shifted to opposite direction by elevated temperatures, increasing with reducing the operation temperature like hydrogen fuel cells, with the values at 600, 550, 500, and 450 °C equaling 0.95, 1.0, 1.03, and 0.98 V, respectively. These results propose that the decomposition of ammonia in the cell probably follows a two‐step process: initial decomposition of ammonia to hydrogen and nitrogen, and subsequent dissolution of hydrogen into Pd anode as follows

(2) $${\mathrm{NH}}_{3}⇌{\mathrm{1/2N}}_{2}+{\mathrm{3/2H}}_{2}$$

(3) $$H_{2}\to {\mathrm{2H}}^{\mathrm{Pd}}$$

Here, H^Pd^ denotes hydrogen dissolves in Pd.

In order to evaluate the effective hydrogen partial pressure in the anode of NH_3_‐fed HMFC at 600 °C, the ammonia decomposition rate was determined by acid–base titration for the 1 m H_2_SO_4_ solution equilibrated with the exhaust gases from the anode of NH_3_‐fed HMFC, indicating that about 65% of NH_3_ feeds were decomposed by reaction (2) with yielding a mixed gas of H_2_/N_2_/NH_3_ = 60/20/20. This value is consistent with the thermodynamic equilibrium data,^30^ since ammonia decomposition is favored at temperatures above ≈200 °C. Based on this analysis, the results of Figure 2b reveal that efficiency of NH_3_ type HMFC is corresponding to that of the H_2_ type using equivalent‐*p*_H2_ hydrogen fuel gases.

### 2.2 Polarization Behavior

**Figure 4:**
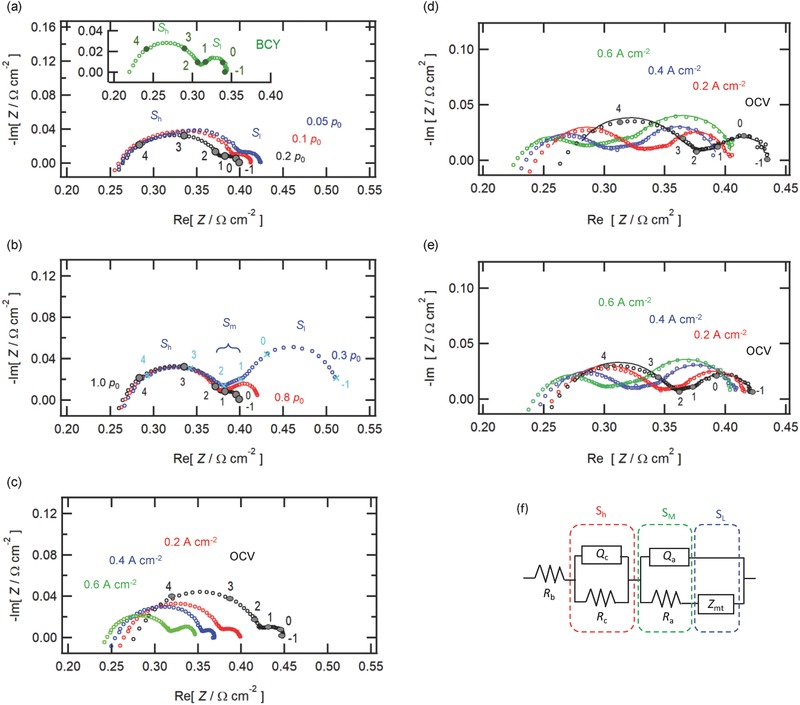
Impedance responses of H_2_ type HMFC (H_2_, Pd | BCY | LSCF, wet air) operating at 200 mA cm^−2^ DC current by changing a) oxygen partial pressure (*p*_O2_) in cathode gas and b) hydrogen partial pressure (*p*_H2_) in anode gas. In (a), the inset shows a reference spectra of BCY‐based HMFC operating in 200 mA cm^−2^ DC current at *p*_O2_ = 0.2 *p*_0_ in the cathode and *p*_H2_ = 1.0 *p*_0_ in the anode.^21^ Responses of HMFC under various DC conditions with c) pure H_2_ fuel, d) NH_3_ fuels, and e) diluted H_2_ fuel (*p*_H2_ = 0.6 *p*_0_). In (d,e), circles show the observed spectra and solid lines show the fitting curves calculated with an equivalent circuit model depicted in Fig. 4f. In (a–e), the decades of the frequencies at each highlighted point are shown.

To provide further verification for the efficient power generation of direct ammonia type HMFC, the interfacial polarization resistances were evaluated by electrochemical impedance spectroscopy. In our previous report, spectra of the BCY‐based HMFC with H_2_ fuels had been deconvoluted by a systematic survey of the electrochemical impedance responses.^21^ The HMFC exposes three distinct arcs in Nyquist plots: relatively large S_h_ arc in a high frequency region at around 10^4^–10^2^ Hz due to the cathode interfacial charge transfer, very small S_m_ arc in a middle frequency region at around 10^2^–10^1^ Hz due to the charge transfer at Pd/electrolyte interfaces and small S_l_ arc in a low frequency region at around 10^1^–10^−1^ Hz related to the hydrogen diffusion‐limited terms for the anodic charge transfer reactions (see the inset of **Figure** **4**a).^21^, ^22^

These spectral features have been replicated well by using an equivalent circuit depicted in Figure 4f, where the Ohmic resistance, *R*_b_, cathodic interfacial charge transfer, (*R*_c_ *Q*_c_), and anodic charge transfer correlated with the mass transfer in Pd bulk, (*Q*_a_(*R*_a_ *Z*_mt_)), are connected in series.^21^ *R* is a resistance and *Q* is a constant phase element representing a time‐dependent capacitance,^32^ with the parallel‐connected *R*_i_ and *Q*_i_ being related to the capacitance *C*_i_ by

(4) $$C_{i}={\left(R_{i}\cdot Q_{i}\right)}^{\mathrm{1/}ni}R_{i}^{-1}$$

*Z*_mt_ denotes the hydrogen mass transfer impedances, which is associated with the perturbation of hydrogen permeation flux across Pd membrane,^33^, ^34^ and is represented with an admittance *Y*_mt_ and a time decay constant *B*_mt_ by

(5) $$Z_{\mathrm{mt}}={\left(Y_{\mathrm{mt}}\sqrt{\left(j\omega \right)}\right)}^{-1}\tanh\left\{B_{\mathrm{mt}}\sqrt{j\omega }\right\}$$

(6) $$B_{\mathrm{mt}}=L/D_{H}^{0.5}$$

*L* is the thickness of membranes, *D*_H_ diffusion coefficient, *j* a square root of −1, and ω angular frequency. Equations (5) and (6) are called Nernst circuit element, which provides the mass transfer resistance, *R*_mt_, and capacitance, *C*_mt_, by following^34^

(7) $$R_{\mathrm{mt}}\;\;=\;\;B_{\mathrm{mt}}\;Y_{\mathrm{mt}}^{-1}$$

(8) $$C_{\mathrm{mt}}\;\;=\;\;Y_{\mathrm{mt}}\;B_{\mathrm{mt}}$$

In H_2_‐fed HMFC,^21^ cathodic *S*_h_ arcs could be represented very well by (*R*_c_ *Q*_c_) elements, and the *R*_c_ thus determined were found to be proportional to *p*_O2_^−1/4^ in cathode gases, indicating that *R*_c_ could be assigned to the interfacial charge transfer reaction controlled by oxide ion transfer at gas‐electrode–electrolyte triple phase boundary zones.^35^ The anode reaction of HMFC can be deconvoluted into three elemental steps: Step (i) hydrogen dissolution, step (ii) diffusion of hydrogen dissolves in Pd and step (iii) oxidation of hydrogen dissolves

(9) $$\mathrm{Step}\;\left(i\right):\;H_{2}\to {\mathrm{2H}}^{\mathrm{Pd}}$$

(10) $$\mathrm{Step}\;\left(\mathrm{ii}\right):\;H^{\mathrm{Pd}}\to H^{\mathrm{int}}$$

(11) $$\mathrm{Step}\;\left(\mathrm{iii}\right):\;H^{\mathrm{int}}\to H^{+\mathrm{int}}+e^{-}$$

Here H^int^ are hydrogen dissolves in Pd/BCY interface, and H^+ int^ is proton in the interface. Based on the theoretical circuit model for the electrochemical hydrogen insertion into Pd membrane electrodes,^34^ anodic S_m_ arcs could be assigned to the charge transfer step at Pd/BCY interfaces (Step (iii)) and S_l_ arc to the hydrogen diffusion in Pd bulk (Step (ii)), and thus the anodic polarization is replicated by (*Q*_a_(*R*_a_ *Z*_mt_)) with parallel‐connected *Q*_a_ and *R*_a_ representing S_m_ arc and *Z*_mt_ expressing S_l_ arc.^21^, ^22^ *R_a_* and *R*_mt_ determined by fitting roughly correlated with *p*_H2_^−1/2^, which is in agreement with the reaction orders of steps (ii) and (iii) with respect to *p*_H2_.^21^, ^22^

Figure 4a,b shows the impedance responses of BZCY‐based HMFCs with adjusting oxygen partial pressure in the cathode ($p_{O_{2}}^{\prime }$) or hydrogen partial pressure in the anode ($p_{H_{2}}^{\prime \prime }$), confirming that the spectral features are essentially same as ones of the BCY‐based HMFC (Figure 4a). Hereafter, prime and double prime denote cathode (La_0.6_Sr_0.4_Fe_0.8_Co_0.2_O_3_, LSCF side) and anode (Pd side), respectively. S_h_ arc is clearly assigned to the contribution of the cathodic interfacial charge transfers, because it increases with decreasing $p_{O_{2}}^{\prime }$ (Figure 4a) and systematically decreases with DC currents, i.e., LSCF being polarized more cathodic (Figure 4c). BZCY‐based HMFC clearly shows the anode contributions of S_l_ and S_m_ arcs, because S_l_ is drastically enlarged and S_m_ as a tail in high frequency side at around 10^2^–10^1^ Hz becomes more evident with reducing $p_{H_{2}}^{\prime \prime }$ in the anode although S_m_ is too small and so unclear by overlapping with S_l_ and S_h_ in $p_{H_{2}}^{\prime \prime }$ ≥ 0.8 *p*_0_ (Figure 4b).

In $p_{H_{2}}^{\prime \prime }$ = 1.0 *p*_0_, anodic impedance arcs are not sensitive to DC current, indicating that anodic overpotentials are much smaller than voltage losses at cathodes or electrolytes albeit with high DC outputs (Figure 4c). However, both anodic arcs (S_l_ and S_m_) of HMFC are much enhanced when $p_{H_{2}}^{\prime \prime }$ is changed from 1.0 *p*_0_ to 0.6 *p*_0_, and these mass transfer‐related polarization arcs remarkably grow up with increasing DC currents at $p_{H_{2}}^{\prime \prime }$ = 0.6 *p*_0_, disclosing that larger DC output potentiates anodic concentration overpotential in the operation with a diluted H_2_ fuel. Impedance responses of NH_3_ type HMFC are identical to those of H_2_ type HMFC operating at the equivalent $p_{H_{2}}^{\prime \prime }$ (0.6 *p*_0_) under any DC conditions, with involving a large anodic S_l_ arc as much as cathodic S_h_ arc and significant growth of anodic S_l_ and *S*_m_ arcs with increasing DC current (Figure 4d), which implies that the anode polarization resistances of NH_3_ type HMFC are almost same as those of the H_2_ type with equivalent $p_{H_{2}}^{\prime \prime }$.

**Figure 5:**
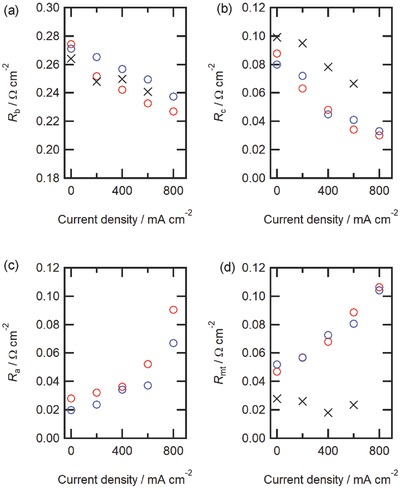
Ohmic and polarization resistances of NH_3_ and H_2_ type HMFCs under various DC conditions at 600 °C, determined by impedance equivalent circuit analysis. a) Electrolyte resistances, *R*_b_, b) cathode polarization resistances, *R*_c_, and c) anode interfacial charge transfer resistances, *R*_a_, and d) hydrogen mass transfer terms *R*_mt_. NH_3_ (○), diluted H_2_ (*p*_H2_ = 0.6 *p*_0_; △), and pure H_2_ (*p*_H2_ = 1.0 *p*_0_; ×).

All impedance responses could be nicely replicated with the equivalent circuit model depicted in Figure 4f regardless of the fuel types and DC current conditions (Figure 4c,d). The fitting parameters are listed in Tables S1–S3 (Supporting Information) and the related resistances are plotted as a function of DC current in **Figure** **5**. At $p_{H_{2}}^{\prime \prime }$ = 1.0 *p*_0_, *S*_m_ arc is much smaller than *S*_h_ and *S*_l_ arcs (Figure 4c), and thus the corresponding spectrum is fitted without including *R*_a_ and *Q*_a_.^21^, ^22^

For H_2_ type HMFC, *R*_mt_ is much smaller than *R*_c_ at any DC conditions in $p_{H_{2}}^{\prime \prime }$ = 1.0 *p*_0_, whereas the anodic resistances given by a sum of *R*_a_ and *R*_mt_ are predominant to the cathodic *R*_c_ in $p_{H_{2}}^{\prime \prime }$ = 0.6 *p*_0_ (Figure 5). Moreover, all resistances of NH_3_ type HMFC are in close agreement with those of the H_2_ type with $p_{H_{2}}^{\prime \prime }$ = 0.6 *p*_0_ in the all DC range, proving that NH_3_ type HMFC can undergo power generation by utilizing hydrogen products via ammonia pyrolysis without large overpotentials for the direct electrooxidation and/or surface poisoning of ammonia chemisorbed. Hence, current‐enhanced anodic polarization resistances of NH_3_‐fed HMFC can be mainly attributed to the concentration overpotentials due to the delay of the hydrogen mass diffusion inside Pd bulk under relatively low $p_{H_{2}}^{\prime \prime }$.

### 2.3 Electronic Properties of BZCY/Pd Oxide‐Metal Junctions

**Figure 6:**
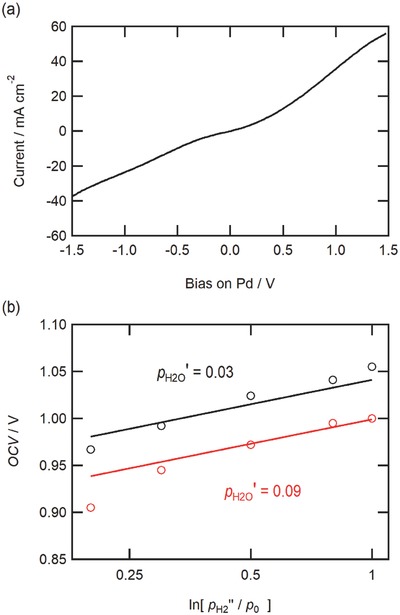
a) *I*–*V* characteristic of the cell with the following configuration: Ar, Pd | BZCY | LSCF, dry air. Here, the bias on Pd metal is controlled. b) OCV of the “pseudo” hydrogen concentration cells, measured at 600 °C with the configuration H_2_/Ar ($p_{H_{2}}^{\prime \prime }$), Pd | BaZr_0.1_Ce_0.7_Y_0.2_O_3−_ *_δ_* | LSCF, H_2_O/O_2_/Ar ($p_{H_{2}O}^{\prime }$, $p_{O_{2}}^{\prime }$). Here, $p_{H_{2}}^{\prime \prime }$ is varied in the range of 0.2 to 1.0 *p*_0_, $p_{H_{2}O}^{\prime }$ is adjusted at 0.03 or 0.09 *p*_0_, and $p_{O_{2}}^{\prime }$ is fixed at 0.001 *p*_0_. Symbols (○) show the observed, and solid lines are calculated by Equation (14). The details of calculation is mentioned in main text.

**Figure 7:**
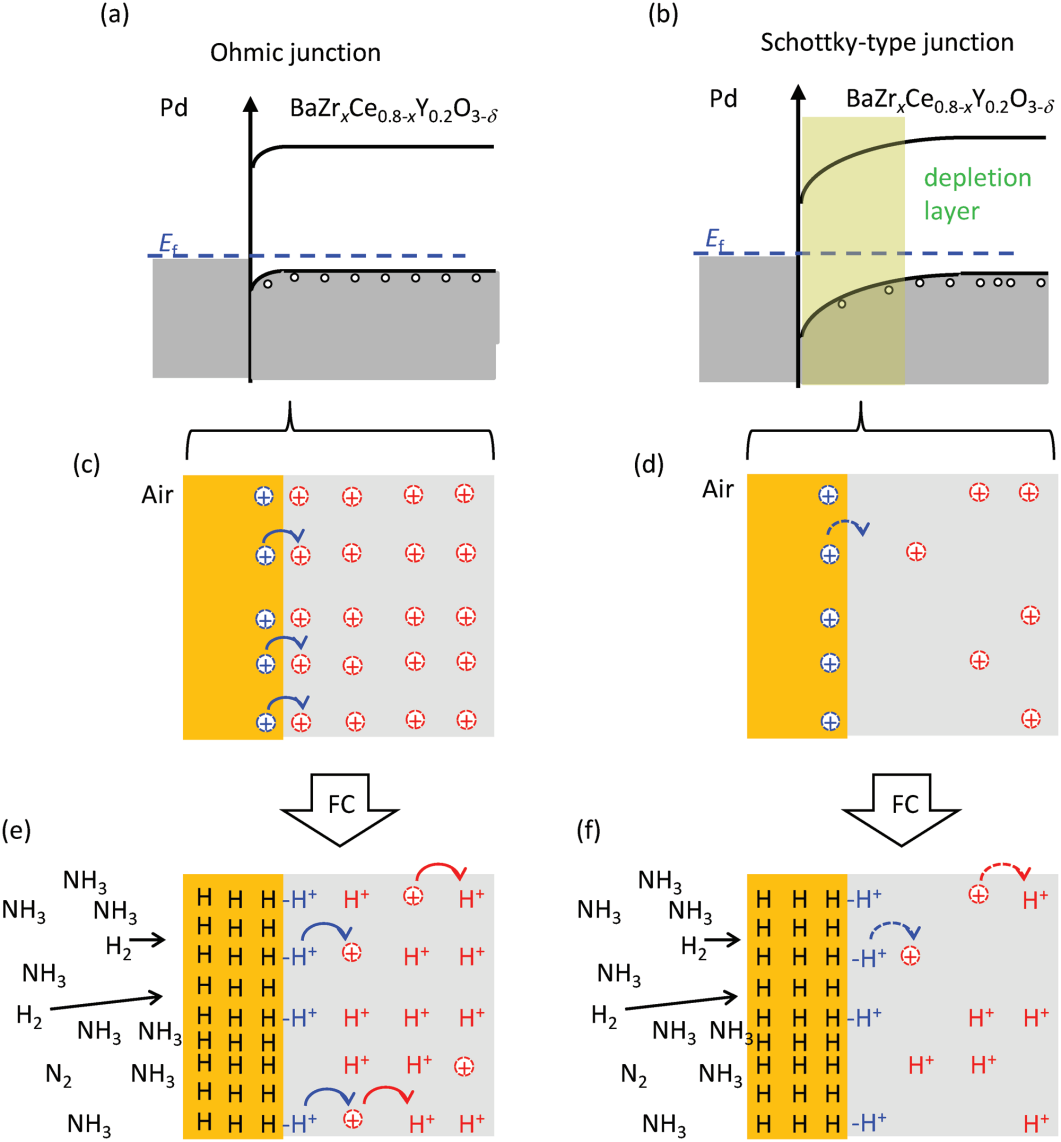
Schematic representations of electronic and thermodynamic properties of BZCY/Pd oxide‐metal junctions featured by a,c,e) Ohmic contact without forming wide interfacial depletion layer or b,d,f) Schottky‐type barrier with growth of wide interfacial depletion layer. a) Flat band alignment of the junctions establishing Ohmic contact and b) band bending of Schottky‐type junction. Hole carrier concentrations in the BZCY/Pd interfacial region under dry atmosphere in the case of c) Ohmic contact or d) Schottky‐type junction. Proton defects concentrations in the interfacial region of fuel cell in the case of e) Ohmic contact or f) Schottky‐type junction. In (e) and (f), blue protons indicate the protonic intermediates formed by anodic reactions at BZCY/Pd interfaces, red arrows represent creation of excess holes by hopping of protons to neighbor sites, and blue arrows represent proton transfer at BZCY/Pd interfaces, driven by hole–proton thermodynamic equilibrium (22). The details are described in main text.

Since BZCY has been reported to show partial hole conductivity in relatively high *p*_O2_ atmosphere,^36^, ^37^ the BZCY/Pd junction essentially acts as a p‐semiconductor/metal junction. It is well known that a potential energy barrier for electron, so called, Schottky barrier, is formed at metal–semiconductor junctions,^38^ where the bands of the p‐semiconductors are bended upward by a contact with metals for alignment of Fermi energy levels, leading to the depletion layer of hole carriers in the semiconductor/metal interfaces (Figure 7b,c).^38^ Such devices tend to show the current rectification with allowing nominal current flow only by applying a positive bias on p‐semiconductor (i.e., forward bias). In order to clarify the electronic properties of the BZCY/Pd junctions, *I–V* characteristics were tested for the cell: dry Ar, Pd | BZCY | LSCF, dry air (**Figure** **6**a). In this case, the main charge carriers are electron holes because of the absence of proton sources in the atmosphere. The devices exhibit symmetric, nonlinear *I*–*V* characteristics without occurrence of remarkable current rectification (Figure 6a), confirming that depletion layer at the BZCY/Pd interfaces is very narrow so that the junction allows a large tunneling current in both bias directions, thereby, establishing Ohmic contact.^38^

In order to evaluate the proton transfer number of HMFC under a presence of oxygen in the cathode side, OCV were measured for pseudo hydrogen concentration cells: H_2_/Ar, Pd | BZCY | LSCF, wet 0.1%‐O_2_/Ar (Figure 6b). Here, $p_{H_{2}}^{\prime \prime }$ in the anode is adjusted in a range of 0.2 to 1.0 *p*_0_ and the humidified, 0.1%‐O_2_ gas ($p_{O_{2}}^{\prime }$ = 0.001 *p*_0_) is supplied to the cathode side. In the general case, the thermodynamic OCV value of such cells can be defined as follows^39^

(12) $$\mathrm{OCV}\;\;=\;\;t_{O}\frac{RT}{4F}\;\mathrm{In}\;\left(\frac{p\prime_{O_{2}}}{p\prime \prime_{O_{2}}}\right)\;\;+\;\;t_{H}\frac{RT}{2F}\mathrm{In}\left(\frac{p\prime_{H_{2}}}{p\prime \prime_{H_{2}}}\right)$$

Here, the prime′ and double prime″ denote cathode (LSCF side) and anode (Pd side), respectively. *t*_O_ and *t*_H_ are the transfer number of oxide ion and protons, *F* a Faradaic constant, and *R* a gas constant. $p_{H_{2}}^{\prime }$ can be defined by coupled with water dissociation reaction as follows

(13) $$p_{H_{2}}^{\prime }=K_{w}p_{H_{2}O}p_{O_{2}}^{\prime }{}^{-1/2}$$

Here, *K*_w_ is an equilibrium constant and *p*_H2O_ water partial pressure. BZCY electrolyte is well known to exhibit mixed oxide ion and proton conduction under fuel cell conditions.^40^ Nonetheless, the contribution of partial oxide ion conduction must be limited in HMFC because the Pd solid electrodes act as a blocking electrode of oxide ion, and thus *t*_O_ can be assumed to be nearly zero. Finally, OCV of the HMFC is given by the following

(14) $$\mathrm{OCV}\;\;=\;\;t_{H}\frac{RT}{2F}\;\mathrm{In}\;\left(\frac{K_{w}p\prime_{H_{2}O}p\prime_{O_{2}}{}^{\;\;\;\;\;-0.5}}{p\prime \prime_{H_{2}}}\right)\;\;=\;\;t_{H}\frac{RT}{2F}\;\mathrm{In}\;\left(\frac{K_{w}p\prime_{H_{\;2}O}p\prime_{O_{2}}{}^{\;\;\;\;\;\;-0.5}}{p_{H_{2}{}^{\;\mathrm{Pd}}}}\right)$$

Effective hydrogen partial pressure at the BZCY/Pd interface ($p_{H2}^{\mathrm{Pd}}$) must equal $p_{H_{2}}^{\prime \prime }$ under OCV condition in order to keep zero hydrogen flux in Pd foils. In both humidified conditions ($p_{H_{2}}^{\prime \prime }$ = 0.03, 0.09 *p*_0_), the observed OCVs are in agreement with the calculated by Equation (14) with *t*_H_ = 1.0 in $p_{H_{2}}^{\prime \prime }$ > 0.4 *p*_0_, whereas they are deviated downward from the calculated in $p_{H_{2}}^{\prime \prime }$ < 0.2 *p*_0_ (Figure 6b), revealing that HMFC would cause hole leakage when the $p_{H_{2}}^{\prime \prime }$ is sufficiently decreased with the cathode exposed to oxygen.

## 3 Discussion

### 3.1 Performance Comparison for Direct Ammonia Type Fuel Cells

The preceding results unequivocally demonstrate that HMFCs exhibit superior fuel cell performances to other fuel cell systems by direct NH_3_ feeds. For direct comparison, electrochemical performances of the direct ammonia type PCFCs and SOFCs reported in recent studies^41^, ^42^, ^43^, ^44^, ^45^, ^46^, ^47^ are summarized in **Table** **2**. To best of our knowledge, the highest *P*_max_ at 600 °C (0.4 W cm^−2^) is achieved by an anode‐supported SOFC comprising 20 µm thick Ce_0.9_Sm_0.1_O_2_ electrolyte and Ni‐Ce_0.9_Sm_0.1_O_2_ cermet anode.^41^ The performances of the NH_3_ type PCFCs are still low and the *P*_max_ is in most cases less than 0.2 W cm^−2^, as listed in Table 2. Apparently, the *P*_max_ of our HMFC is higher than the champion data, revealing that polarization resistances of NH_3_ type HMFCs is much lower than those of the PCFCs and SOFCs with porous cermet anodes.

**Table 2:** Summary of the recent literatures for NH_3_‐type PCFCs and SOFCs operating at 600 °C

Electrolyte	Anode cermet	OCV [V]	*P*_max_@600 °C [mW cm^−2^]	Ref.
BaCe_0.8_Gd_0.2_O_3_ (10 µm)	Ni‐Ce_0.8_Gd_0.2_O_2_	1.1	150	44
BaCe_0.8_Gd_0.2_O_3_ (10 µm)	Ni‐Ce_0.9_Gd_0.1_O_2_	1.0	210	46
BaZr_0.1_Ce_0.7_Y_0.2_O_3_ (20 µm)	Ni‐BaZr_0.1_Ce_0.7_Y_0.2_O_3_	1.15	190	42
BaCe_0.9_Y_0.1_O_3_ (60 µm)	Ni‐BaCe_0.75_Y_0.25_O_3_	1.07	165	43
Zr_0.9_Sc_0.1_O_2_ (20 µm)	Fe/Ni‐Zr_0.9_Y_0.1_O_2_	1.1	240	45
Ce_0.9_Sm_0.1_O_2_ (20 µm)	Ni‐Ce_0.9_Gd_0.1_O_2_	0.9	410	41
Ce_0.9_Sm_0.1_O_2_ (50 µm)	Ni‐Ce_0.9_Sm_0.1_O_2_	0.88	170	47
BaZr_0.1_Ce_0.7_Y_0.2_O_3_ (1 µm)	Pd foil	0.98	580	This work

The largest voltage losses in HMFC are assigned to the Ohmic losses mainly due to electrolyte resistances (*R*_b_; Figure 5a). 1 µm thick BZCY thin films possess *R*_b_ of about 0.27 Ω cm^−2^ at OCV conditions, and the corresponding proton conductivity σ becomes 2.7 × 10^−4^ S cm^−2^, which is two orders of magnitude lower than the values of the sintered pellets (4.9 × 10^−2^ S cm^−2^).^36^ The deteriorated σ of the films must be owing to the precipitates in a grain boundary region, deviation of the stoichiometry, and high resistivity of grain boundaries, and so on.^48^, ^49^ *R*_b_ of HMFC is remarkably decreased with DC currents, indicating that the proton conductivity of electrolyte is enhanced by an applied field. Several authors reported on the modification of ion carrier profiles by biasing in ion‐conducting thin films sandwiched by ion‐blocking electrodes.^50^, ^51^ In such case, the cationic or anionic ion carriers tend to be accumulated in the region beneath oppositely charged electrodes and depleted in another side by the drifts under applied fields, and thus the overall ion conductivity of films is varied by the modified ion profiles. Because the anode of fuel cells is more positively biased if DC current is higher, i.e., cell voltage is lower, the mobile O^2−^ anions must be accumulated in the BZCY layer in vicinity of Pd blocking electrodes under DC conditions, altogether with protons for charge compensation, which may improve the proton conductivity by the increment of proton concentrations in the interfacial layers.

Previously, we have demonstrated that BCY‐based HMFC has significantly lowered cathode polarization resistances *R*_c_, which is 20 times smaller than that of the anode‐supported type PCFCs comprising same BCY/LSCF cathode configuration.^21^, ^52^ *R*_c_ of the current BZCY‐based HMFC is lower than 0.1 Ω cm^−2^ in both H_2_ and NH_3_ types at any DC conditions (Figure 5), which is similar as those of BCY‐HMFC.^21^ The mechanism for lowering cathodic interfacial resistances would be reported in next paper.

The impedance fitting analysis clearly demonstrated that a large concentration overpotential takes place at the anode by using NH_3_ or diluted H_2_ fuels particularly in high DC current conditions owing to the limited hydrogen mass transfer in Pd bulk (Figure 5c,d). The fundamental studies on the anodic polarization behavior of ammonia fuel cells have been still quite a few. Yang et al. reported that NH_3_ type PCFC made of a BaCe_0.75_Y_0.25_O_3−_ *_δ_* electrolyte with a Ni‐BaCe_0.75_Y_0.25_O_3−_ *_δ_* cermet anode had the anodic overpotential of 0.08 V under 0.06 A cm^−2^ at 600 °C.^53^ The corresponding anodic resistance is calculated to be 1.2 Ω cm^−2^, which is at least one order of magnitude larger than the sum of *R*_a_ and *R*_mt_ of the HMFC operating at 0.6 A cm^−2^, revealing that the anode reaction of NH_3_‐type HMFC is significantly faster than the reaction at the Ni‐BZCY‐gas triple phase boundaries of NH_3_ type PCFC despite the former going on with mediated via mass diffusion in metal anode.

In order to output 1 A cm^−2^ DC currents, hydrogen flux (*J*_H_) in Pd is required to 1.05 × 10^−5^ mol cm^−2^ s^−1^, which can be given by the following with hydrogen permeability ϕ and thickness *l* 54

(15) $$J_{H}=\varphi l^{-1}{\left(p\prime \prime_{H_{2}}-p_{H_{2}}^{\mathrm{Pd}}\right)}^{0.5}=\varphi l^{-1}\Delta p_{H_{2}}^{0.5}$$

Here Δ*p*_H2_ is the *p*_H2_ gap between both Pd edges, which is corresponding to $p_{H_{2}}^{\prime \prime }$ – $p_{H2}^{\mathrm{Pd}}$. By using ϕ = 4 × 10^−8^ mol cm^−1^ s^−1^ Pa^−0.5^ 55 and *l* = 30 µm, the Δ*p*_H2_ is calculated to about 13 kPa for the *J*_H_ value, clarifying that $p_{H2}^{\mathrm{Pd}}$ is still as high as 0.45 *p*_0_ under operating in 1.0 A cm^−2^ DC currents by NH_3_ fuels, i.e., $p_{H_{2}}^{\prime \prime }$ = 0.6 *p*_0_. This analysis strongly suggests that NH_3_‐type HMFC can generate sufficiently high DC powers by utilizing only pyrolytic H_2_ products due to the high hydrogen permeability of Pd anode. It is clearly concluded that HMFC is viable fuel cells enabling efficient power generation by direct ammonia feeds at around 600 °C.

### 3.2 Proton Transfer at Oxide‐Metal Junctions

In BZCY, the proton carriers are in equilibrium with moisture in the atmosphere as follows^56^, ^57^

(16) $$V_{O}^{\cdot \cdot }\;\;+\;\;H_{2}O\to {\mathrm{2H}}_{i}^{•}\;\;+\;\;O_{O}^{\times }$$

The corresponding equilibrium constant (*K*_1_) is given by

(17) $$K_{1}=\left[H_{i}^{•}\right]{\;}^{2}\;\;\left[O_{O}^{\times }\right]\;\;{\left[V_{O}^{\cdot \cdot }\right]}^{\;-1}p_{H_{2}O}^{-1}$$

Here, according to Kröger–Vink notations, $V_{O}^{\cdot \cdot }$, $O_{O}^{\times }$, and $H_{i}^{\cdot }$represent oxygen vacancies, lattice oxygens, and proton defects, respectively, and [] denotes the concentration of each specie. The hole carriers, h^•^, are incorporated into BZCY by the following defect reactions

(18) $$V_{O}^{\cdot \cdot }\;\;+\;{\mathrm{1/2O}}_{2}\to O_{O}^{\times }\;\;+\;\;{\mathrm{2h}}^{•}$$

(19) $$K_{2}\;\;=\;\;\left[O_{O}^{\times }\right]\;\;{\left[h^{\cdot }\right]}^{2}\;\;{\left[V_{O}^{\cdot \cdot }\right]}^{\;\mathrm{–1}}p_{O_{2}}^{\mathrm{–1/2}}$$

Proton and hole carriers are in equilibrium by the following reactions

(20) $$H_{2}O\;\;+\;\;{\mathrm{2h}}^{•}\to {\mathrm{2H}}_{i}^{•}\;\;+\;\;{\mathrm{1/2O}}_{2}$$

(21) $$K_{3}\;\;=\;\;{\left\{\left[H_{i}^{•}\right]/\left[h^{\cdot }\right]\right\}}^{2}p_{O_{2}}^{\mathrm{1/2}}p_{H_{2}O}^{-1}$$

*K*_2_ and *K*_3_ are equilibrium constants of Equations (18) and (20), respectively. By coupled with water dissociation reaction, *K*_3_ of Equation (21) is rewritten as a function of *p*_H2_ as follows

(22) $$K_{3}\;\;=\;\;{\left\{\left[H_{i}^{•}\right]/\left[h^{\cdot }\right]\right\}}^{2}K_{w}p_{H_{2}}^{-1}$$

Ohmic contacts between Pd and BZCY (Figure 6a) would not be convenient to block the electron (hole) charge transfer across the interface. In fact, the HMFC increases hole transfer number when *p*_H2_ in the anode is significantly lowered (<0.2 *p*_0_) with increasing [h^•^] in relation to equilibrium (22) (Figure 6b). Nevertheless, Equation (22) suggests that the absence of wide depletion layer at the junction is advantageous for the anodic charge transfer reactions of HMFC. In BZCY electrolyte, protons’ jumping to neighbor sites leaves an excess hole behind for the local charge neutrality (**Figure** **7**e,f). Therefore, moving away of protons from the interfacial sites gives rise to unequilibrated [$H_{i}^{\cdot }$]/[h^•^] ratio within the interfacial regions, which exerts incorporation of the proton intermediates formed on the Pd surface into BZCY layer in order to recover the [$H_{i}^{\cdot }$]/[h^•^] thermodynamic equilibration (22) (Figure 7e). In case of the “Ohmic” BZCY/Pd junctions, the [$H_{i}^{\cdot }$] in the interfacial region of BZCY electrolyte is not depleted according to the hole–proton equilibrium (22) because Ohmic contact accepts relatively high [h^•^] in the interfacial region (Figure 7c,e), whereas allowable [$H_{i}^{\cdot }$] in the interfacial region of the Schottky‐type junction would be much lower than that of the Ohmic contact because of lowering [h^•^] in the interfaces (Figure 7d,f). In fact, it has been reported that the SrZr_0.9_Y_0.1_O_3_/Pd junction which forms Schottky‐type potential barrier with wide depletion layer at the interface shows lessened conductivity because the proton concentrations in the interfacial layer becomes smaller than those of the SrZr_0.9_Y_0.1_O_3_ bulk.^58^, ^59^ These imply that the proton incorporation via “Ohmic” BZCY/Pd junction is much faster than that via Schottky‐type junction due to the relatively large acceptable concentrations of hydrogen defects ([$H_{i}^{\cdot }$]) in the interfacial region (Figure 7e). Although the physical model mentioned above is purely analytical, however, it strongly suggests that Ohmic contact between BZCY electrolytes and Pd anodes is indispensable for the fast charge transfer reaction mediated via proton–hole exchanges at the BZCT/Pd interfaces.

## 4 Conclusion

It is demonstrated that hydrogen membrane fuel cells based on the BZCY‐Pd oxide‐metal junction enables efficient power generation by direct feed of NH_3_ fuels at intermediate temperatures. The corresponding anode reactions are found to proceed via the thermal decomposition of NH_3_ and the subsequent oxidation of the hydrogen products. In HMFCs, Pd metal anodes can efficiently capture pyrolytic hydrogen products from the mixed fuel gases with subsequently transferring them to the reaction interface due to the excellent hydrogen permeability. Moreover, Ohmic contact of BZCY/Pd junction allows to accommodate large amounts of protons in the interfacial region and thus promotes the incorporation of proton from Pd into BZCY according to the hole–proton thermodynamic equilibrium, thereby accelerating the anodic charge transfer reaction. Accordingly, NH_3_‐fed HMFCs possess significantly lowered anodic polarization resistance, which lead the superior fuel cell performances to the corresponding SOFCs and PCFCs with gaining stable power outputs of 0.58 W cm^−2^ at 600 °C. It is concluded that the HMFC comprising highly conducting ceramic electrolytes and hydrogen sorption alloys is greatly advantageous for power generation with not only NH_3_ but also other hydrogen carriers by utilizing efficiently their pyrolytic hydrogen products at intermediate temperatures. The current results exert a great influence on the direction in the developments of the next‐generation intermediate‐temperature fuel cells.

## 5 Experimental Section

Previously, a BCY thin film was successfully fabricated by RF cosputtering technique with BaCe_0.8_Y_0.2_O_3−_ *_δ_* and CeO_2_ double targets.^21^, ^22^ BZCY thin films were also fabricated by the same method with BaCe_0.8_Y_0.2_O_3−_ *_δ_* and ZrO_2_ targets. Phase purity and crystallinity of the deposited films were checked by X‐ray diffraction patterns with a RIGAKU diffractometer (RIGAKU Rint2000). The scanning electron microscopy was conducted with JEOL JSM‐7100F. The chemical composition of BCY thin films was determined by WDX with a JEOL JXA‐8530F. The scanning transmission electron microscopy (STEM) was carried out in a HITACHI HD‐2000. The specimens for TEM observation were prepared by a focused ion beam microfabrication (HITACHI FB‐2100).

The HMFCs were fabricated by depositing a BZCY thin film of 1 µm thickness on a Pd foil (0.05 × 12 × 12 mm, Tanaka Co.). The foil was polished with alumina particles (1.0 µm diameter) and was cleaned by sonication in acetone and pure water before deposition. LSCF button electrode (7 mm∅) was screen‐printed on the surfaces of the BZCY films as a porous cathode by using a commercial LSCF paste (FuelcellMaterials) with subsequent heating by a heat‐gun for 2 min.

The performances of the HMFCs were evaluated by measuring the current–voltage (*I–V*) relation and electrochemical impedance spectra at elevated temperatures. The specimen was sealed in a specially designed sample holder with a mica gasket (FuelcellMaterials). Both cathode and anode were contacted with Pt meshes, and a thermocouple was placed in close proximity to the cell to obtain temperature data as accurate as possible. Normally, dry H_2_ or NH_3_ gases were fed to the Pd anode side at a flow rate of 100 cm^3^ min^−1^, and wet air gas was fed to the cathode side at a rate of 100 cm^3^ min^−1^. The wet gas (*p*_H2O_ = 3 kPa) was prepared by passing the gases through water at 25 °C. The *p*_H2_ and *p*_O2_ in anode and cathode gases, respectively, were adjusted with Ar balance gases. Electrochemical impedance spectroscopy and *I–V* characteristics were obtained by using a Solartron 1260/1287 system in the frequency range of 10^6^ to 0.1 Hz with AC amplitude of 30 mV under several DC bias voltages. The effective hydrogen partial pressures in the anode gases of NH_3_‐fed HMFC were determined by acid–base titration for the 1 m H_2_SO_4_ solution equilibrated with exhaust gases from the anode sides of NH_3_‐fed HMFC. For this, anode exhausts were bubbled in H_2_SO_4_ solution for 1 h during power generation under 0.75 V potentiostatic condition at 600 °C, and the resultant solutions were titrated with 0.1 m NaOH standard solutions and a pH meter (Horiba D‐71LAB).

## Conflict of Interest

The authors declare no conflict of interest.

## Supporting information

Supplementary

## References

[1] A. Klerke , C. H. Christensen , J. K. Norskov , T. Vegge , J. Mater. Chem. 2008, 18, 2304.

[2] M. Kitano , Y. Inoue , Y. Yamazaki , F. Hayashi , S. Kanbara , S. Matsuishi , T. Yokoyama , S.‐W. Kim , M. Hara , H. Hosono , Nat. Chem. 2012, 4, 934. 23089869 10.1038/nchem.1476

[3] F. Schüth , R. Palkovits , R. Schlögl , D. S. Su , Energy Environ. Sci. 2012, 5, 6278.

[4] R. Lan , K. J. T. S Irvin , S. Tao , Int. J. Hydrogen Energy 2012, 37, 1482.

[5] R. Schlögl , Angew. Chem., Int. Ed. 2003, 42, 2004. 10.1002/anie.200301553 12746811

[6] A. Afif , N. Radenahmad , Q. Cheok , S. Shams , J. H. Kim , A. K. Azad , Renewable Sustainable Energy Rev. 2016, 60, 822.

[7] K. Lui , R. Yan , G. Meng , X. Liu , Ionics 2009, 15, 115.

[8] N. Maffei , L. Pelletier , J. P. Carland , A. McFarlan , J. Power Sources 2008, 175, 221.

[9] L. Pelletier , A. McFarlan , N. Maffei , J. Power Sources 2005, 145, 262.

[10] L. Yang , C. Zuo , S. Wang , Z. Cheng , M. Liu , Adv. Mater. 2008, 20, 3280.

[11] G. Taillades , J. Dailly , M. Taillades‐Jacquin , F. Mauvy , A. Essouhmi , M. Marrony , C. Lalanne , S. Fourcade , D. J. Jones , J. C. Grenie , J. Rozière , Fuel Cells 2010, 10, 166.

[12] L. Yang , S. Wang , X. Lou , M. Liu , Int. J. Hydrogen Energy 2011, 36, 2266.

[13] N. Nasani , D. Ramasamy , S. Mikhalev , A. V. Kovalevsky , D. P. Fagg , J. Power Sources 2015, 278, 582.

[14] R. Strandbakke , V. A. Cherepanov , A. Y. Zuev , D. S. Tsvetkov , C. Argirusis , G. Sourkouni , S. Prünte , T. Norby , Solid State Ionics 2015, 278, 120.

[15] D. Pergolesi , E. Fabbri , A. D'Epifanio , E. D. Bartolomeo , T. Tebano , S. Sanna , S. Licoccia , G. Balestrino , E. Traversa , Nat. Mater. 2010, 9, 846. 20852619 10.1038/nmat2837

[16] L. Yang , C. Zuo , S. Wang , Z. Cheng , M. Liu , Adv. Mater. 2008, 20, 3280.

[17] Y. Guo , Y. Lin , R. Ran , Z. Shao , J. Power Sources 2009, 193, 400.

[18] Y. Yamazaki , R. Hernandez‐Sanchez , S. M. Haile , Chem. Mater. 2009, 21, 2755.

[19] E. Fabbri , D. Pergolesi , E. Traversa , Chem. Soc. Rev. 2010, 39, 4355. 20818453 10.1039/b902343g

[20] N. Ito , M. Iijima , K. Kimura , S. Iguchi , J. Power Sources 2005, 152, 200.

[21] Y. Aoki , S. Kobayashi , T. Yamaguchi , E. Tsuji , H. Habazaki , K. Yashiro , T. Kawada , T. Ohtsuka , J. Phys. Chem. C 2016, 120, 15976.

[22] Y. Aoki , T. Yamaguchi , E. Tsuji , H. Habazaki , J. Electrochem. Soc. 2017, 154, F577.

[23] N. Ito , S. Aoyama , T. Matsui , S. Matsumoto , H. Matsumoto , T. Ishihara , J. Power Sources 2008, 185, 922.

[24] J. Kim , S. Sengodan , G. Kwon , D. Ding , J. Shin , M. Liu , G. Kim , ChemSusChem 2014, 7, 2811. 25146887 10.1002/cssc.201402351

[25] K. Bae , D. Y. Jang , H. J. Choi , D. Kim , J. Hong , B.‐K. Kim , J.‐H. Lee , J.‐W Son , J. H. Shim , Nat. Commun. 2017, 8, 14553. 28230080 10.1038/ncomms14553 PMC5331335

[26] C. Duan , J. Tong , M. Shang , S. Nikodemski , M. Sanders , S. Ricote , A. Almonsoori , R. O'Hayre , Science 2015, 349, 1325. 10.1126/science.aab3987 26217064

[27] K. Bae , H.‐S. Noh , D. Y. Jang , H. J. Hong , H. Kim , K. J. Yoon , J.‐H. Lee , B.‐K. Kim , J. H. Shim , J.‐W Son , J. Mater. Chem. A 2016, 4, 6395.

[28] T. Sato , T. Inoue , D. Ichinose , H. Funakubo , K. Uchiyama , Jpn. J. Appl. Phys. 2016, 55, 02BC19.

[29] A. Oda , T. Okumura , T. Higuchi , ECS Trans. 2013, 57, 1019.

[30] A. H. White , W. Melville , J. Am. Chem. Soc. 1905, 27, 373.

[31] A. Fuerte , R. X. Valenzuela , M. J. Escudero , L. Daza , J. Power Sources 2009, 192, 170.

[32] A. Bierberle‐Hütter , M. Sogaard , H. L. Tuller , Solid State Ionics 2006, 177, 1969.

[33] C. Montella , J. Electroanal. Chem. 2000, 480, 150.

[34] J. S. Chen , J.‐P. Diard , R. Durand , C. Montella , J. Electroanal. Chem. 1996, 406, 1.

[35] F. He , T. Wu , R. Peng , C. Xia , J. Power Sources 2009, 194, 263.

[36] S. Ricotte , N. Bonanos , G. Caboche , Solid State Ionics 2009, 177, 900.

[37] S. Ricotte , N. Bonanos , H. J. Wang , R. Haugsrud , Solid State Ionics 2011, 185, 11.

[38] S. M. Sze , K. K. Ng , Physics of Semiconductor Devices, John Wiley & Sons, New York, USA 2007.

[39] T. Norby , Solid State Ionics 1988, 23–30, 1586.

[40] H. Iwahara , T. Yajima , T. Hibino , H. Ushida , J. Electrochem. Soc. 1993, 140, 1687.

[41] G. Meng , C. Jiang , J. Ma , Q. Ma , X. Liu , J. Power Sources 2007, 173, 189.

[42] Y. Lin , R. Ran , Y. Guo , W. Zhou , R. Cai , J. Wang , Z. Shao , Int. J. Hydrogen Energy 2010, 35, 2637.

[43] J. Yang , A. F. S. Molouk , T. Okanishi , H. Muroyama , T. Matsui , K. Eguchi , ACS Appl. Mater. Interfaces 2015, 7, 7406. 25804559 10.1021/acsami.5b01048

[44] L. Zhang , W. Yang , J. Power Sources 2008, 179, 92.

[45] L. Liu , K. Sun , X. Wu , X. Li , M. Zhang , N. Zhang , X. Zhou , Int. J. Hydrogen Energy 2010, 37, 10857.

[46] K. Xie , Q. Ma , B. Lin , Y. Jiang , J. Gao , X. Liu , G. Meng , J. Power Sources 2007, 170, 38.

[47] Q. Ma , R. Peng , L. Tian , G. Meng , Electrochem. Commun. 2006, 8, 1791.

[48] J. H. Shim , T. M. Gür , F. B. Prinz , Appl. Phys. Lett. 2008, 92, 253115.

[49] D. Pergolesi , E. Fabbri , E. Traversa , Electrochem. Commun. 2010, 12, 977.

[50] Y. Aoki , C. Wiemann , V. Feyer , H.‐S. Kim , M. Scneider , H. Ill‐Yoo , M. Martin , Nat. Commun. 2014, 4, 3473. 10.1038/ncomms4473 24632885

[51] Y. Gil , O. M. Umurchan , Y. Tsur , I. Riess , Solid State Ionics 2008, 179, 24.

[52] E. Fabbri , S. Licoccia , E. Traversa , E. D. Wachsman , Fuel Cells 2009, 9, 128.

[53] J. Yang , T. Akagi , T. Okanishi , H. Muroyama , T. Matsui , K. Eguchi , Fuel Cells 2015, 15, 390.

[54] L. Cornaglia , J. Munera , E. Lombardo , Int. J. Hydrogen Energy 2015, 40, 3423.

[55] G. L. Holleck , J. Phys. Chem. 1970, 74, 503.

[56] H. Iwahara , Solid State Ionics 1996, 86–88, 9.

[57] K. D. Kreuer , Annu. Rev. Mater. Res. 2003, 33, 333.

[58] H. Matsumoto , Y. Furuya , S. Okada , T. Tanji , T. Ishihara , Sci. Technol. Adv. Mater. 2007, 8, 531.

[59] H. Matsumoto , Y. Furuya , S. Okada , T. Tanji , T. Ishihara , Electrochem. Solid‐State Lett. 2007, 10, P11.

