# Supplementary material for: High‐Efficiency Direct Ammonia Fuel Cells Based on BaZr0.1Ce0.7Y0.2O3− δ/Pd Oxide‐Metal Junctions
Source: Glob Chall. 2017 Dec 14;2(1):1700088. doi: 10.1002/gch2.201700088 (PMC6607173; doi:10.1002/gch2.201700088)
Supplement: Supplementary file 1 — Supplementary [file GCH2-2-1700088-s001.pdf]

Copyright WILEY-VCH Verlag GmbH & Co. KGaA, 69469 Weinheim, Germany, 2017.

Global Challenges
Open Access

Supporting Information

for *Global Challenges,* DOI: 10.1002/gch2.201700088

High-Efficiency Direct Ammonia Fuel Cells Based on $BaZr_{0.1}Ce_{0.7}Y_{0.2}O_{3-\delta}$/Pd Oxide-Metal Junctions

Yoshitaka Aoki, Tomoyuki Yamaguchi, Shohei Kobayashi, Damian Kowalski, Chunyu Zhu, and Hiroki Habazaki*

Copyright WILEY-VCH Verlag GmbH & Co. KGaA, 69469 Weinheim, Germany, 2016.

Supporting Information

High efficiency direct ammonia fuel cells based on $BaZr_{0.1}Ce_{0.7}Y_{0.2}O_{3-\delta}$/Pd oxide-metal junctions

Yoshitaka Aoki, Tomoyuki Yamaguchi, Shohei Kobayashi, Damian Kowalski, Chunyu Zhu,*

Hiroki Habazaki

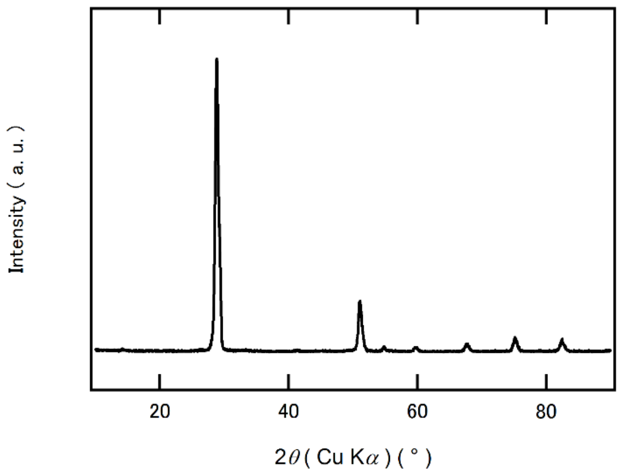

Figure S1. XRD patterns of BZCY thin films deposited by rf cosputtering with BCY and ZrO_2 double targets in optimal conditions as listed in Table 1.

Table S1 Fitting parameters of impedance spectroscopy of HMFC (p_{H2} = 1.0 atm) at 600°C under various DC conditions. The original spectra are shown in Figure 4(c).

	OCV	200 mA cm^{-2}	400 mA cm^{-2}	600 mA cm^{-2}	800 mA cm^{-2}
R_b / Ω cm^2	0.25	0.22	0.21	0.20	0.19
R_c / Ω cm^2	0.15	0.085	0.060	0.042	0.039
n_c	0.75	0.79	0.82	0.85	0.86
C_c / F cm^{-2}	2.8×10^{-4}	6.2×10^{-4}	8.7×10^{-4}	1.1×10^{-3}	1.4×10^{-3}
Y_{mt} / S s$^{-0.5}$	0.42	0.44	0.49	0.48	0.51
B_{mt} / s$^{-0.5}$	0.42	0.39	0.39	0.36	0.40
R_{mt} / Ω cm^2	0.040	0.035	0.032	0.031	0.031
C_{mt} / F cm^{-2}	16	18	20	20	20
D_H / cm^2 s^{-1}	1.4×10^{-4}	1.6×10^{-4}	1.6×10^{-4}	1.9×10^{-4}	1.5×10^{-4}

Table S2 Fitting parameters of impedance spectroscopy of HMFC (p_{H2} = 0.6 atm) at 600°C under various DC conditions. The original spectra are shown in Figure 4(e).

	OCV	200 mA cm^{-2}	400 mA cm^{-2}	600 mA cm^{-2}	800 mA cm^{-2}
R_b / Ω cm^2	0.271	0.266	0.257	0.249	0.237
R_c / Ω cm^2	0.080	0.072	0.045	0.041	0.033
n_c	0.87	0.87	0.92	0.97	0.97
C_c / F cm^{-2}	3.3×10^{-4}	3.7×10^{-4}	5.5×10^{-4}	7.3×10^{-4}	1.1×10^{-3}
R_a / Ω cm^2	0.019	0.024	0.034	0.037	0.067
n_a	0.72	0.71	0.75	0.66	0.66
C_a / F cm^{-2}	0.087	0.069	0.055	0.043	0.034
Y_{mt} / S s$^{-0.5}$	0.51	0.45	0.47	0.49	0.51
B_{mt} / s$^{-0.5}$	0.66	0.65	0.67	0.59	0.54
R_{mt} / Ω cm^2	0.052	0.057	0.073	0.081	0.10
C_{mt} / F cm^{-2}	16	14	11	9.5	7.1
D_H / cm^2 s^{-1}	5.7×10^{-5}	5.9×10^{-5}	5.5×10^{-5}	7.2×10^{-5}	8.8×10^{-5}

Table S3 Fitting parameters of impedance spectroscopy of NH_3-fed HMFC at 600°C under various DC conditions. The original spectra are shown in Figure 4(d).

	OCV	200 mA cm^{-2}	400 mA cm^{-2}	600 mA cm^{-2}	800 mA cm^{-2}
R_b / Ω cm^2	0.274	0.252	0.242	0.233	0.226
R_c / Ω cm^2	0.087	0.063	0.048	0.034	0.030
n_c	0.91	0.91	0.94	1	1
C_c / F cm^{-2}	2.6×10^{-4}	3.9×10^{-4}	5.9×10^{-4}	9.1×10^{-4}	1.2×10^{-3}
R_a / Ω cm^2	0.028	0.033	0.036	0.052	0.090
n_a	0.75	0.71	0.65	0.66	0.66
C_a / F cm^{-2}	0.050	0.042	0.036	0.036	0.024
Y_{mt} / S s$^{-0.5}$	0.50	0.42	0.34	0.28	0.19
B_{mt} / s$^{-0.5}$	0.59	0.59	0.58	0.53	0.49
R_{mt} / Ω cm^2	0.047	0.057	0.068	0.088	0.11
C_{mt} / F cm^{-2}	16	14	11	8.9	6.6
D_H / cm^2 s^{-1}	7.2×10^{-5}	7.2×10^{-5}	7.4×10^{-5}	8.9×10^{-5}	9.7×10^{-5}
